# Technical Optimization Strategies for Amyloid PET Under Challenging Acquisition Conditions: A Comprehensive Narrative Review

**DOI:** 10.3390/diagnostics16132033

**Published:** 2026-06-29

**Authors:** Luca Camoni, Francesco Dondi, Agata Pietrzak, Roberto Rinaldi, Francesca Tomasoni, Michela Cossandi, Silvia Lucchini, Gian Luca Viganò, Luigi Spiazzi, Francesco Bertagna

**Affiliations:** 1 Allied Health and Social Care Professions Directorate, ASST Spedali Civili di Brescia, University of Brescia, 25123 Brescia, Italy; luca.camoni@unibs.it; 2 Nuclear Medicine, ASST Spedali Civili di Brescia, University of Brescia, 25123 Brescia, Italy; francesco.bertagna@unibs.it; 3 Nuclear Medicine Department, Greater Poland Cancer Centre, 61-866 Poznan, Poland; apietrzak@ump.edu.pl; 4 Electroradiology Department, Poznan University of Medical Sciences, 61-866 Poznan, Poland; 5 Nuclear Medicine, ASST Spedali Civili di Brescia, 25123 Brescia, Italy; roberto.rinaldi@asst-spedalicivili.it (R.R.); francesca.tomasoni@asst-spedalicivili.it (F.T.); michela.cossandi@asst-spedalicivili.it (M.C.); silvia.lucchini@asst-spedalicivili.it (S.L.); 6 Clinical Engineering, ASST Spedali Civili di Brescia, 25123 Brescia, Italy; gianluca.vigano@asst-spedalicivili.it; 7 Health Physics Department, ASST Spedali Civili di Brescia, 25123 Brescia, Italy; luigi.spiazzi@asst-spedalicivili.it

**Keywords:** Alzheimer’s disease, amyloid, positron emission tomography, acquisition-time reduction, low-count imaging, motion correction, artificial intelligence, deep learning, image restoration

## Abstract

Amyloid PET is increasingly used to confirm cerebral amyloid burden, but standard acquisition may be compromised by head motion, limited patient cooperation, reduced effective counts, premature scan termination, or non-repeatable imaging conditions. This comprehensive narrative review used a structured evidence-mapping approach in accordance with SANRA quality criteria. A structured literature search was performed in PubMed/MEDLINE, Scopus, and Web of Science up to 15 March 2026. Eligible studies included clinical, phantom, or hybrid studies addressing acquisition-time reduction, injected-activity reduction or low-count imaging, motion correction, or artificial intelligence-based image enhancement. Findings were synthesized narratively because of substantial heterogeneity in tracers, scanners, protocols, reconstruction methods, populations, comparators, and endpoints. Sixteen studies were included. Moderate reductions in acquisition time or effective counts generally preserved semiquantitative performance, whereas visual interpretation became more vulnerable under more aggressive reductions, borderline amyloid status, or reduced image quality. Artificial intelligence-based restoration improved image-quality metrics and supported interpretation of short- or low-count acquisitions, but evidence remained model-specific. Motion correction was supported by one amyloid-specific [^18^F]flutemetamol PET/CT study and should be interpreted as a potentially useful but under-replicated strategy. Current evidence supports cautious, tracer-, scanner-, reconstruction-, and task-specific optimization under challenging acquisition conditions rather than universal protocol reduction or direct generalization to motion-prone, poorly cooperative, or non-repeatable acquisition scenario. Reduced protocols, artificial intelligence-based restoration, and motion correction should remain locally validated supportive strategies, not substitutes for standard acquisition.

## 1. Introduction

Alzheimer’s disease (AD) is the most common cause of dementia. According to Global Burden of Disease 2019 estimates, 57.4 million people worldwide were living with dementia in 2019 and this number is expected to rise to 152.8 million by 2050, mainly because of population aging and growth [1,2].

Alongside this growth, the introduction of anti-amyloid therapies has required confirmation of amyloid presence, using the amyloid positron emission tomography (PET) and cerebrospinal fluid biomarkers to identify patients eligible for treatment [3,4,5,6]. Therefore, amyloid PET is playing a key role in AD, particularly in the evolving landscape of diagnosis, therapeutic eligibility, and appropriate biomarker use [7,8]. The three main [^18^F]-labeled tracers used in clinical practice, namely [^18^F]florbetaben (Neuraceq; Life Molecular Imaging GmbH, Berlin, Germany), [^18^F]florbetapir (Amyvid; Eli Lilly and Company, Indianapolis, IN, USA), and [^18^F]flutemetamol (Vizamyl; GE Healthcare AS, Oslo, Norway), enable in vivo visualization of cortical amyloid-beta (Aβ) deposition. When interpreted together with the clinical and neuropsychological information, amyloid PET can improve diagnostic assessment [9,10].

At the same time, the technical reliability of amyloid PET is influenced by interdependent variables, including injected activity and the uptake time, acquisition duration, scanner characteristics, reconstruction and post-processing protocol, and finally—motion correction [9,11,12]. Under standard conditions, these variables are controlled through validated acquisition and reconstruction protocols. However, standard acquisition may be challenged by head motion, reduced effective counts, premature scan interruption, difficulty maintaining a stable supine position, communication barriers, anxiety, tremor, pain, or limited patient cooperation. These conditions may occur more frequently in patients with cognitive impairment. When present, they may increase the risk of motion artifacts, reduce image quality, decrease reader confidence, and, in severe cases, compromise the diagnostic value of the examination [9,13,14].

In this review, the term “challenging acquisition conditions” refers to situations in which amyloid PET is at increased risk of technical degradation, including motion artifacts, reduced effective acquisition time, low-count reconstruction, poor image quality, reduced reader confidence, or non-repeatable scanning. This terminology is intended to keep the focus on acquisition-related vulnerability while acknowledging that such conditions may be particularly relevant in patients with cognitive impairment, poor cooperation, or difficulty maintaining a stable head position [9,13,14]. Challenging acquisition conditions may also arise from radiopharmaceutical and workflow-related factors. Although uncommon, delayed tracer delivery, radioactive decay of residual activity, extravasation, reduced injected or effective activity, and the need to reorganize patient scheduling may reduce available counts or complicate acquisition timing [15,16]. In such settings, the clinically relevant question is not whether the standard protocol can be simplified, but whether validated or potentially applicable technical strategies can preserve image quality, quantitative reliability, and diagnostic interpretability when optimal acquisition conditions are compromised and reacquisition is not feasible.

Several technological developments have begun to address these issues. Digital PET systems based on silicon photomultipliers (SiPM), improved timing resolution, and optimized reconstruction algorithms may support shorter acquisitions or lower-count imaging in selected settings [17,18,19]. In parallel, artificial intelligence (AI), deep learning (DL), and image restoration methods have been investigated as tools for improving image quality from short-duration, low-count, or technically degraded PET data [20,21,22,23]. Motion-correction approaches represent a further complementary strategy because they address spatial inconsistency during acquisition rather than count limitation alone.

Most available amyloid PET optimization studies were not designed specifically in fragile, poorly compliant, or non-cooperative populations. Instead, they evaluated technical surrogate conditions, including shortened acquisition, simulated low-count imaging, ultra-low-dose protocols, AI-based image restoration, and motion correction. Therefore, the potential relevance of these strategies to patients at higher risk of technically compromised acquisitions remains inferential. This limitation is important because technical feasibility in cooperative research cohorts does not automatically imply clinical validity in patients with motion, poor cooperation, borderline amyloid status, or non-repeatable examinations. The aim of this comprehensive narrative literature review was to summarize and critically discuss technical strategies for amyloid PET optimization under challenging acquisition conditions using [^18^F]florbetaben, [^18^F]florbetapir, and [^18^F]flutemetamol. Four domains were considered: acquisition-time reduction, injected-activity reduction or simulated low-count imaging, AI-based image enhancement or restoration, and motion correction. The purpose of this review was not to establish a universal reduced protocol or to claim direct validation in non-cooperative populations, but to map the available technical evidence, clarify its limitations, and identify which strategies may be cautiously considered when standard acquisition is compromised, non-repeatable, or at high risk of technical failure.

## 2. Materials and Methods

### 2.1. Review Design

This article was designed as a comprehensive narrative literature review with a structured evidence-mapping approach. The review focused on technical strategies for optimizing brain amyloid PET under challenging acquisition conditions.

The structure of the review was aligned with the quality domains proposed by SANRA for narrative review articles, including justification of the topic’s importance, clear statement of aims, transparent description of the literature search, scientific reasoning, presentation of evidence level, and reporting of relevant endpoint data [24].

In this manuscript, the term “comprehensive” refers to the breadth of the literature mapping across the main [^18^F]-labeled amyloid PET radiopharmaceuticals used in clinical practice, namely [^18^F]florbetaben, [^18^F]florbetapir, and [^18^F]flutemetamol; the principal technical optimization domains relevant to challenging acquisition conditions; and clinical, phantom, and hybrid study designs.

### 2.2. Literature Search

A structured literature search was performed across three databases: PubMed/MEDLINE, Scopus, and Web of Science. The search identified studies on brain amyloid PET evaluating technical strategies for examination optimization using [^18^F]florbetaben, [^18^F]florbetapir, or [^18^F]flutemetamol. The last search was performed on 15 March 2026.

The search strategy combined terms related to PET imaging, amyloid pathology, the target radiopharmaceuticals, and technical optimization approaches. The core search string was adapted to each database as follows: (“PET” OR “positron emission tomography” OR “PET/CT” OR “PET/MR”) AND (“amyloid” OR “amyloid beta” OR “Aβ”) AND (“florbetaben” OR “florbetapir” OR “flutemetamol” OR “Neuraceq” OR “Amyvid” OR “Vizamyl”) AND (“acquisition time” OR “scan time” OR “scan duration” OR “shortened acquisition” OR “dose reduction” OR “low dose” OR “low-count” OR “list-mode” OR “artificial intelligence” OR “deep learning” OR “machine learning” OR “image enhancement” OR “image restoration” OR “motion correction”).

The search was centered on technical optimization strategies that may affect amyloid PET acquisition under challenging conditions, including shortened acquisition, reduced-count imaging, image restoration, and motion correction. This approach was chosen to map the available technical evidence across the main domains relevant to acquisition vulnerability, image-quality preservation, quantitative reliability, and diagnostic interpretability.

### 2.3. Eligibility Criteria

Original clinical studies, phantom studies, and hybrid clinical/phantom studies were considered eligible when they evaluated at least one technical optimization strategy relevant to amyloid PET imaging.

The main domains of interest were acquisition-time reduction, injected-activity reduction or simulated low-count imaging, artificial intelligence-based image enhancement or restoration, and motion correction.

Studies were eligible if they evaluated brain amyloid PET using [^18^F]florbetaben, [^18^F]florbetapir, or [^18^F]flutemetamol and directly examined at least one technical strategy intended to preserve or improve image quality, diagnostic interpretability, or quantitative reliability under shortened, reduced-count, motion-degraded, or AI-restored conditions. Eligible comparators included standard-duration PET, full-count PET, standard injected activity, conventional reconstruction, uncorrected images, or expert visual interpretation.

Studies were excluded if they focused exclusively on diagnostic accuracy of amyloid PET without technical optimization, non-amyloid tracers, non-brain PET applications, animal-only experiments, editorials, narrative reviews, conference abstracts without full text, or studies in which the technical intervention could not be separated from unrelated methodological aims.

### 2.4. Study Selection and Data Extraction

Data extraction was performed by reviewing the full text of the selected articles. For each study, general characteristics were collected, including first author, year of publication, radiopharmaceutical, injected activity, population, and sample size. Technical and imaging-related information was also retrieved, including PET system, acquisition duration, reconstruction or post-processing strategy, comparator or reference condition, and the main quantitative and qualitative findings reported by the authors. Titles and abstracts were screened independently by two reviewers (LC, FD). Full texts of potentially eligible studies were then assessed for inclusion according to the predefined criteria. Disagreements were resolved by consensus, and, when necessary, by consultation with a third reviewer.

Given the heterogeneity of the available literature in terms of radiopharmaceutical, scanner technology, acquisition protocol, reconstruction method, study population, comparator, and reported endpoints, findings were summarized narratively. The reviewed studies were grouped according to the main technical optimization strategy evaluated: acquisition-time reduction, injected-activity reduction or low-count imaging, artificial intelligence-based image enhancement or restoration, and motion correction. The synthesis focused on clinically interpretable domains rather than pooled effect estimates.

### 2.5. Domain-Level Evidence-Maturity Appraisal

No formal study-level risk-of-bias assessment was performed because this article was designed as a comprehensive narrative literature review, and because the included evidence comprised heterogeneous study designs, including clinical studies, phantom or hybrid evaluations, simulated reduced-count analyses, AI-based image-restoration studies, and motion-correction investigations.

Instead, a domain-level evidence-maturity appraisal was performed in line with the SANRA-oriented presentation of evidence level, and reporting of relevant endpoint data. For each technical domain, evidence maturity was qualitatively appraised by considering the number of amyloid PET studies available, directness of the evidence to amyloid PET, study design, use of real versus simulated acquisition conditions, tracer and scanner coverage, availability of visual and semiquantitative endpoints, consistency of findings, external or prospective validation, and clinical transferability.

This domain-level appraisal was used to distinguish technical feasibility from clinical readiness and was not intended as a formal GRADE rating or study-level risk-of-bias assessment. This domain-level appraisal is reported in Supplementary Table S2.

### 2.6. Use of Generative Artificial Intelligence

The authors used a generative AI tool to assist with language refinement and rephrasing during manuscript preparation (GPT-5.5 Thinking; OpenAI OpCo, LLC, San Francisco, CA, USA). All scientific content, study-eligibility decisions, extracted data, results, and interpretations were independently reviewed, verified, and finalized by the authors based on the original articles.

## 3. Results

### 3.1. Literature Selection and Evidence Map

The database search identified 1486 records. After removal of 482 duplicates, 1004 titles and abstracts were screened. Of these, 965 records were excluded because they did not address amyloid PET technical optimization, used non-target tracers, were not brain PET studies, or were reviews, editorials, or conference abstracts. Thirty-nine full-text articles were assessed for eligibility. Twenty-three articles were excluded: nine addressed early, middle-phase, or alternative acquisition windows, or predictive analyses without a primary focus on technical optimization; ten compared protocols, software, or radiotracers without a separable amyloid PET optimization strategy or included radiotracers outside the scope of this review; and four focused primarily on the computed tomography (CT) or magnetic resonance (MR) component rather than on amyloid PET optimization. The literature selection process is summarized in Supplementary Figure S1 and Table S1.

Sixteen studies met the inclusion criteria and were included in the final narrative synthesis.

The included studies have evaluated technical strategies aimed at preserving image quality, quantitative reliability, and diagnostic interpretability in amyloid positron emission tomography (PET) performed with [^18^F]florbetaben, [^18^F]florbetapir, or [^18^F]flutemetamol. The evidence was organized into four technical domains: acquisition-time reduction, injected-activity reduction or simulated low-count imaging, artificial intelligence-based image enhancement or restoration, and motion correction.

Six studies evaluated acquisition-time reduction; three examined injected-activity reduction or simulated low-count imaging; six investigated artificial intelligence-, deep learning-, or machine learning-based image enhancement or restoration; and one assessed motion correction. The reviewed studies were heterogeneous in terms of radiopharmaceutical, population, scanner technology, acquisition protocol, reconstruction strategy, comparator, and outcome measures. The synthesis reports both study-level endpoint data and domain-level interpretation. Quantitative endpoints included standardized uptake value ratio (SUVR), Centiloid values, receiver operating characteristic metrics, area under the curve (AUC), sensitivity, specificity, image-quality metrics, and agreement measures. Qualitative endpoints included visual classification, reader confidence, image-quality scores, and brain amyloid plaque load (BAPL)-based interpretation.

Table 1, Table 2, Table 3 and Table 4 summarize the main study characteristics, technical strategies, comparators, and clinically relevant findings across the four technical domains.

### 3.2. Acquisition-Time Reduction

Six studies assessed shortened acquisition protocols for amyloid PET imaging [25,26,27,28,29,30]. Overall, quantitative discrimination was generally preserved across reduced acquisition durations, whereas visual assessment showed greater performance degradation when acquisition time was markedly shortened.

Tiepolt et al. [25] assessed shortened [^18^F]florbetaben PET acquisitions reconstructed at 5, 10, and 20 min. Composite SUVR remained significantly higher in patients with AD than in healthy volunteers at all scan durations (*p* < 0.001), without meaningful within-group differences across durations. Cohen’s d values were 4.53, 3.93, and 4.76 for the 5, 10, and 20 min reconstructions, respectively. Visual sensitivity and specificity remained unchanged at 80% and 96%, respectively, while inter-reader agreement ranged from κ = 0.89 to κ = 0.94.

Otani et al. [26] evaluated 10, 20, and 30 min acquisition protocols. Visual classification was identical across all three acquisition durations. Among (Aβ)-negative participants, SUVR values remained stable across acquisition conditions, with a median fluctuation of 0.4 ± 0.7%. Among Aβ-positive participants, SUVR was influenced by both acquisition duration and scan start time. With 20 min acquisitions, a 5 min shift in scan start time produced SUVR fluctuations of 1.2–1.8%. Variability was reduced when the scan was centered at the midpoint of 105 min after injection.

Wagatsuma et al. [27] compared 5, 10, and 20 min reconstructions. Neither composite SUVR (cSUVR) nor regional SUVR (rSUVR) showed relevant differences across scan durations, and correlations between durations were extremely high (Spearman R = 0.999). Quantitative discrimination remained strong, with AUC values of 0.92, 0.89, and 0.99 for the 5, 10, and 20 min reconstructions, respectively. Mean inter-reader agreement was κ = 0.788.

Xie et al. [28] evaluated shortened acquisition protocols in PET/MR settings. Quantitative classification based on an SUVR threshold of 1.41 was fully preserved down to 6 min. At 4 min, sensitivity, specificity, positive predictive value (PPV), and negative predictive value (NPV) were 97%, 92%, 97%, and 92%, respectively, with a further performance decline at 2 and 1 min. Visual classification was also fully preserved at 6 min. At 4 min, one case became indeterminate, while visual performance decreased more clearly at 2 and 1 min.

Trinh et al. [29] assessed a larger [^18^F]florbetaben cohort and found stable global SUVR and Centiloid values from 5 to 20 min, with no relevant differences between acquisition durations. Using BAPL as the reference standard, ROC and AUC values were 0.99 at 5 min, 0.98 at 10 min, 0.99 at 15 min, and 0.99 at 20 min, respectively. For 5 min acquisitions, the optimal global Centiloid threshold was 21.86, yielding a sensitivity of 0.95 and a specificity of 0.97, respectively. Diagnostic confidence was similar between 5 and 20 min acquisitions.

Yang et al. [30] evaluated shortened acquisitions using a high-performance digital PET/computed tomography (PET/CT) system. Composite cortex SUVR maintained perfect quantitative discrimination from 10 min down to 30 s, with an AUC of 1.00 across all tested durations. Visual classification was unchanged at 10, 2, and 1 min, whereas specificity decreased at 45 and 30 s. Image quality at 2 min was comparable to that at 10 min, while 1 min and 45 s acquisitions still provided diagnostically usable images.

Taken together, these findings suggest that moderate shortening is technically feasible, but ultra-short protocols remain scanner-dependent and visually vulnerable in borderline or very short acquisitions.

### 3.3. Dose Reduction and Low-Count Imaging

Three studies examined injected-activity reduction or simulated low-count imaging [31,32,33]. Quantitative classification was largely preserved with moderate count reduction.

Joshi et al. [31] reported that injected activities of 111 and 370 MBq of [^18^F]florbetapir yielded no significant differences in SUVR at 50–60 min after injection (*p* = 0.27). In a separate test–retest cohort, mean cortical SUVR variability was 2.4 ± 1.41% in patients with AD and 1.5 ± 0.84% in controls, with an overall intraclass correlation coefficient (ICC) of 0.99. Visual classification was unchanged across injected activities, although image-quality scores were lower at 111 MBq.

Herholz et al. [32] used bootstrap simulation to assess [^18^F]florbetapir count reduction from 100% to 10% of the original dataset. The overall cortex-to-cerebellum coefficient of variation ratio increased from 0.009 to 0.025. In the modeled Fleisher cohort, ROC AUC changed only marginally, from 0.881 to 0.878, and sensitivity/specificity at the 1.17 threshold shifted from 82.0%/77.3% to 81.8%/77.1%, respectively. The main effect of count reduction was a broader uncertainty interval, which widened from 1.06–1.19 to 1.03–1.23.

Young et al. [33] assessed simulated count reduction separately for [^18^F]flutemetamol and [^18^F]florbetaben. For [^18^F]flutemetamol, AUC remained 0.96 across count levels; for [^18^F]florbetaben, AUC ranged from 0.94 to 0.96. At 12.5% of the original count level, overall SUVR and Centiloid changes remained below 1.5% for both tracers. Intra-reader agreement remained high for both tracers, although the [^18^F]florbetaben subgroup within the 25% gray zone showed lower agreement.

Overall, low-count robustness was stronger for semiquantitative classification than for visual confidence, and simulation-based evidence should not be considered equivalent to prospective low-activity imaging.

### 3.4. AI/DL/ML-Based Image Enhancement and Restoration

Six studies addressed AI-, DL-, or ML-based enhancement or restoration of short-duration or ultra-low-count amyloid PET images [34,35,36,37,38,39]. Overall, these approaches improved image-quality metrics and preserved diagnostic classification more effectively than unprocessed short-duration or low-count reconstructions.

Chen et al. (2019) [34] evaluated convolutional neural network (CNN)-based enhancement of simulated 1% low-count [^18^F]florbetaben PET images. The PET + MR model achieved the highest peak signal-to-noise ratio (PSNR) in 39 of 40 datasets, together with the best structural similarity index measure (SSIM) and the lowest root mean square error (RMSE) across all datasets. Compared with full-count PET, SUVR bias was lower for PET + MR images (0.04; 95% confidence interval [CI], −0.26 to 0.33). By contrast, raw 1% low-count PET images were unreadable in most assessments.

Chen et al. (2021) [35] assessed DL-based enhancement of effective ultra-low-count PET/MR images corresponding to approximately 2.2% of the standard injected activity. Enhancement significantly improved PSNR, SSIM, and RMSE compared with raw ultra-low-count PET. Classification of amyloid status against standard-count PET reached 97.2% accuracy for enhanced actual ultra-low-count images and 91.7% for enhanced sampled images. All enhanced images met non-inferiority criteria for clinical image quality.

Chen et al. (2022) [36] compared three enhancement strategies for ultra-low-count amyloid PET imaging. All three approaches significantly improved PSNR, SSIM, and RMSE compared with ultra-low-count PET, with no meaningful differences between simultaneous and non-simultaneous MR inputs. The enhanced image pairs received concordant interpretations in 31 of 32 cases.

Lee et al. [37] evaluated DL-based restoration of shortened amyloid PET acquisitions. Normalized root mean square error (NRMSE) decreased and PSNR increased as scan duration lengthened, and the three-slice model consistently outperformed the single-slice model. AUC for amyloid positivity remained between 0.98 and 0.98 across 1 to 5 min scans, respectively. Image quality was poor at 1 min, acceptable at 2–3 min, and good to excellent at 4–5 min.

Jeong et al. [38] assessed generative adversarial network (GAN)-restored 2 min PET images. GAN-restored images showed better PSNR, SSIM, and NRMSE than raw 2 min PET images, with a mean SUVR difference of −0.035 relative to raw 20 min PET. Across three physicians, average BAPL-based reading performance reached 89.1% accuracy, 91.3% sensitivity, and 83.3% specificity. In Turing tests, clinicians could not reliably distinguish synthetic from real PET images.

Peng et al. [39] evaluated MCDNet-based denoising of 5 min amyloid PET scans. MCDNet-2 achieved the best noise reduction performance, with an NRMSE of 0.0941, SSIM of 0.9557, and PSNR of 34.93. It also obtained the highest subjective image-quality score and classified Aβ-positive and Aβ-negative scans with 100% accuracy, whereas raw and Gaussian-filtered 5 min images reached only 84% accuracy.

Across AI studies, image restoration improved similarity metrics and classification consistency, but evidence remained model-specific and only partly externally validated.

### 3.5. Motion Correction

Only one study addressed motion correction. Park et al. [40] applied data-driven head-motion correction to 108 [^18^F]flutemetamol PET/CT scans acquired over 20 min. Quantitative analysis was feasible in 95 of 108 scans. The median difference in uptake ratio between conventional and motion-corrected PET was 0.06 in scans without visible head motion and 0.10 in scans with visible head motion (*p* = 0.016). Motion correction increased the proportion of good-quality scans from 72–76% to 95–97%, eliminated poor-quality scans, improved inter-reader agreement from κ = 0.81 to κ = 0.88, and changed the final interpretation in 11 of 108 scans.

## 4. Discussion

This review summarizes the available evidence on technical strategies that may preserve amyloid PET image quality, quantitative reliability, and diagnostic interpretability when standard acquisition conditions are compromised. The reviewed literature supports four technical domains: acquisition-time reduction, injected-activity reduction or simulated low-count imaging, AI-based image enhancement or restoration, and motion correction. These strategies address different technical vulnerabilities and should be considered complementary. Across domains, the evidence remains uneven: acquisition-time reduction has the closest support for clinical translation, whereas reduced-count imaging, AI-based restoration, and motion correction remain more dependent on simulated data, model-specific validation, or limited amyloid-specific replication.

Importantly, most included studies did not directly enroll patients selected because of frailty, poor cooperation, or motion-prone behavior. Instead, they evaluated technical surrogate conditions, including shortened acquisition, simulated reduced-count imaging, AI-based restoration, and motion correction. The findings should therefore be interpreted as evidence on acquisition-related technical vulnerability rather than as direct validation in fragile or non-cooperative populations.

Indeed, a recurrent finding across acquisition-time and injected-activity reduction studies was the greater apparent stability of semiquantitative endpoints compared with visual interpretation alone. SUVR, Centiloid values, ROC-derived thresholds, and region-based classification were generally preserved over a wider range of reduced acquisition durations or reduced counts than reader-based confidence and visual specificity [25,26,27,28,29,30,31,32,33].

This divergence between quantitative and visual endpoints is clinically relevant. Semiquantitative measures average signal across predefined regions and reference tissues, thereby reducing random noise. Visual interpretation depends on gray matter/white matter contrast, focal cortical uptake, artifacts, and reader confidence. Visual reading is therefore the stricter boundary when protocols are shortened, especially in borderline or equivocal scans. The available evidence does not support a single reduced protocol applicable to all amyloid PET examinations.

For [^18^F]florbetaben, acquisition-time studies found that 5 min imaging at standard injected activity preserved visual and quantitative performance under tested conditions [25,29]. Trinh et al. showed that, despite minimal SUVR and Centiloid bias between 5and 20 min acquisitions, a small number of category shifts occurred when conventional Centiloid cut-offs were applied [29]. This supports the need for acquisition-duration-specific threshold calibration in shortened [^18^F]florbetaben protocols. Clinically, shortened [^18^F]florbetaben imaging should therefore be considered a potentially valid locally validated protocol, not a direct substitute for standard-duration acquisition without recalibrated decision thresholds.

For [^18^F]florbetapir, the evidence was broader but more heterogeneous. Real and simulated injected-activity reduction studies suggest quantitative robustness across moderate count reductions, whereas acquisition-time studies indicate that 5–6 min may represent a feasible lower range in conventional, mixed (conventional/digital scanner), or PET/MR settings, depending on scanner performance and the diagnostic endpoint used [27,28,29,31,32]. Ultra-short [^18^F]florbetapir acquisitions of approximately 1–2 min appear technically promising using a latest-generation PET/CT scanner, but these findings should be interpreted as scanner- and reconstruction-specific and should not be transferred to other scanners, reconstruction protocols, or clinical populations without local validation [30].

For [^18^F]flutemetamol, the evidence supports a more conservative approach. A 10 min acquisition preserved image quality and diagnostic performance in the available study. SUVR was more sensitive to scan-start timing and acquisition conditions, especially in Aβ-positive cases [26]. This point is relevant because [^18^F]flutemetamol has a lower injected activity than [^18^F]florbetaben and [^18^F]florbetapir, potentially reducing the margin for acquisition shortening [26,33].

Injected-activity reduction studies showed a similar pattern. Moderate reduction, up to 50%, generally remained within a stable quantitative range, whereas instability became more visible at lower count levels, particularly around classification thresholds or grey-zone scans [31,32,33].

These findings require careful interpretation because much of the amyloid PET injected-activity reduction evidence comes from list-mode simulation [32,33]. These simulations allow controlled within-subject comparisons. They do not fully reproduce true low-activity acquisition. True low-activity studies may differ in detector background, random coincidences, count-rate behavior, patient motion, attenuation correction, scatter correction, and workflow. Amyloid PET dose reduction should therefore be treated as technically plausible. It should not be treated as interchangeable with standard-activity imaging without tracer-, scanner-, and reconstruction-validation [33].

Scanner technology should be interpreted as a modifier of the feasible reduction range rather than as a binary determinant of success or failure. Several amyloid PET studies showing moderate protocol reduction were performed on conventional systems, suggesting that moderate shortening is not necessarily exclusive to digital SiPM-based PET [25,26,29,33].

At the same time, the most aggressive reductions in amyloid PET were reported on more advanced platforms, particularly systems with high sensitivity, improved timing resolution, and optimized reconstruction [28,30]. Digital SiPM-based systems may therefore extend the lower boundary of feasible acquisition, especially when protocols are pushed toward ultra-short durations or more extreme count-limited conditions. However, hardware alone does not establish diagnostic safety. Clinical performance remains co-determined by tracer kinetics, injected activity, uptake timing, reconstruction algorithm, post-processing, quantitative threshold calibration, and the prevalence of borderline cases [26,27,28,30].

A further point is that reduced acquisition or reduced-activity protocols may deviate from approved product information, institutional protocols, or multicenter trial acquisition standards. Even when technically feasible, such adaptations should not be presented as equivalent to standard protocols unless they have been locally validated and accepted within the clinical governance framework of the imaging center. In clinical trials, longitudinal follow-up, treatment eligibility assessment, or borderline amyloid status, adherence to validated acquisition and reconstruction standards remains particularly important.

From a practical nuclear medicine perspective, technical optimization should begin before image reconstruction. Patient preparation, explanation adapted to cognitive status, comfortable positioning, head fixation, minimization of environmental stress, careful venous access, documentation of injected and residual activity, and verification of uptake and acquisition timing remain essential. In patients or examinations at increased risk of technical degradation, the most clinically valuable strategy may be to combine preventive measures with flexible reconstruction of list-mode data, rather than relying on post-processing alone. Reduced acquisition protocols should therefore be embedded within a quality-control workflow that records motion, effective scan duration, injected activity, residual activity, uptake time, reconstruction parameters, and any deviation from the standard protocol.

AI-based restoration addresses a different part of the problem. Acquisition-time and activity-reduction studies test how far raw or conventionally reconstructed PET can be reduced. AI restoration evaluates whether degraded PET images can be brought closer to a standard-time or standard-activity reference. In the included AI studies, deep learning improved objective image-quality metrics. Several studies also restored visual readability and semiquantitative agreement in count-limited images [34,35,36,38,39]. Multimodal PET/MR approaches appeared particularly effective in ultra-low-count [^18^F]florbetaben settings, likely because anatomical information can guide image restoration [34,35,36]. PET-only approaches are more directly applicable to routine PET/CT workflows and showed promising restoration of short-time acquisitions, but their broader applicability remains dependent on multicenter validation, scanner variability, reconstruction differences, motion conditions, and domain-shift robustness [38,39].

The current AI studies should be read as rescue or support evidence. They do not justify replacing standard acquisition protocols by themselves. Their most plausible role is in short acquisitions, count-limited list-mode recovery, ultra-low-dose experimental protocols, and non-repeatable scans with poor image quality. Most amyloid PET AI studies in this review were published between 2019 and 2022, and they belong largely to an early phase of AI application in amyloid PET, mainly using CNN, U-Net, residual-learning, or GAN architectures [34,35,36,38,39].

More recent neurological PET applications using generative AI, diffusion models, transformer-based approaches, and vendor-integrated deep-learning reconstruction tools indicate rapid methodological development in this field [20,21,22,23,24].

Motion correction represents a distinct optimization strategy. Shortened acquisition and AI restoration primarily address count limitation and image noise, whereas motion correction addresses spatial inconsistency during acquisition. This distinction is particularly relevant because head motion may degrade amyloid PET even when injected activity, uptake window, and acquisition duration are otherwise standard [40]. In the available [^18^F]flutemetamol motion-correction study, image quality improved after correction, poor-quality scans were eliminated, reader concordance increased, and quantitative uptake-ratio differences were greater in patients with detected motion [40]. These findings suggest that motion correction may have clinical relevance beyond cosmetic post-processing, although the evidence remains limited to a single amyloid-specific clinical study and should be generalized cautiously across tracers, scanners, reconstruction algorithms, shortened acquisitions, and low-count protocols. Although amyloid-specific evidence for motion correction remains limited to one clinical study, broader neurological PET data provide contextual support for its technical rationale. In brain [^18^F]fluorodeoxyglucose PET, patient motion has been shown to degrade spatial resolution and introduce regional quantification bias, particularly through emission–attenuation misalignment [41]. Data-driven or markerless approaches suggest that motion correction can be implemented with limited additional burden and may preserve visual and quantitative agreement in clinical brain PET workflows [42,43,44]. Similar relevance has been reported in neurodegenerative tau PET, where correction improved image sharpness and quantitative stability in [^18^F]MK6240 imaging [45,46].

The four technical domains considered in this review should therefore be regarded as complementary. Shortened acquisition may reduce the time during which motion can occur, but it cannot correct motion that has already happened. AI restoration can improve low-count image, but it may not reliably recover spatially misplaced uptake if the input or training target is blurred or misregistered. Motion correction directly targets spatial degradation, but does not solve low-count noise by itself. Future amyloid PET studies should evaluate these approaches both separately and in combination. Future protocols should report motion magnitude, visual confidence, reader agreement, SUVR or Centiloid effects, and interactions between motion correction, reduced-time acquisition, low-count reconstruction, and AI-restored protocols.

### 4.1. Study Limitations

Several limitations should be considered when interpreting this review. First, the clinical scenario that motivated the review—amyloid PET performed under challenging acquisition conditions—was not directly represented in most included cohorts. Most studies evaluated technical surrogate conditions, such as shortened acquisition, simulated reduced-count imaging, AI-based restoration, or motion correction, often in cooperative, retrospectively selected, or technically controlled datasets. Therefore, the findings should be interpreted as technical evidence relevant to compromised acquisitions rather than as direct clinical evidence in motion-prone, poorly cooperative, or non-repeatable acquisition scenarios.

The included studies differed substantially in tracer, scanner technology, acquisition protocol, reconstruction method, injected activity, uptake window, population, comparator, and endpoint selection. This heterogeneity precluded quantitative pooling and supported a narrative, domain-based synthesis rather than a meta-analysis.

Several studies were retrospective, single-center, simulation-based, or based on selected cohorts. Some included clearly separated Alzheimer’s disease and healthy control groups, which may not fully reflect memory-clinic populations, where amyloid burden is often borderline, equivocal, or clinically discordant. This limits the direct transferability of diagnostic thresholds, visual confidence, and reduced-protocol performance to routine clinical practice.

Several injected-activity reduction or low-count studies used list-mode resampling rather than prospective reduced-activity acquisition. These approaches allow controlled within-subject comparisons but may not fully reproduce true low-activity imaging, because detector background, random coincidences, count-rate behavior, patient motion, attenuation correction, scatter correction, and workflow may differ.

The AI-based studies were mainly early proof-of-concept investigations. External validation was often limited, and most endpoints focused on image similarity, noise reduction, quantitative agreement, or classification consistency. Fewer studies tested prospective reader performance, diagnostic impact, reporting safety, failure modes, or implementation under real-world clinical conditions. Broader neurological PET studies using generative models, transformer-based methods, or PET-to-PET translation remain contextual and cannot replace amyloid-specific validation.

Amyloid-specific motion-correction evidence remains limited. Only one clinical study in this review directly tested motion correction in amyloid PET, using [^18^F]flutemetamol PET/CT. Extension to [^18^F]florbetaben, [^18^F]florbetapir, PET/MR, shortened acquisitions, low-count protocols, or different motion patterns requires separate validation. In addition, changes in final amyloid interpretation after motion correction should be interpreted as diagnostic impact or classification shifts, not as proof of improved diagnostic accuracy in the absence of an independent reference standard.

Finally, no formal study-level risk-of-bias assessment was performed. Instead, evidence maturity was appraised qualitatively at domain level to account for the heterogeneity of study designs, technical interventions, endpoint types, and validation status. This approach supports interpretation of clinical readiness and validation gaps, but it does not replace a formal risk-of-bias assessment or a prospective validation study.

### 4.2. Clinical Implications and Future Applications

In clinical practice, reduced amyloid PET protocols should be regarded as study-specific operating conditions rather than interchangeable alternatives to standard imaging. This applies both to moderate acquisition shortening on conventional PET/CT systems and to ultra-short acquisitions on high-performance digital PET/CT systems. The available evidence supports technical feasibility only under the specific tracer, scanner, injected activity, uptake window, reconstruction method, and endpoint conditions in which each protocol was tested. Local validation should therefore include visual confidence, semiquantitative stability, borderline scan behavior, motion sensitivity, acquisition-specific quantitative thresholds, and agreement with the local clinical reading workflow [28,29,30,33,40]. An additional practical implication concerns the potential recovery of motion-degraded list-mode acquisitions. If head motion affects only part of the scan, reconstruction of motion-free frames while excluding corrupted segments may be considered only when the retained data remain comparable to a locally validated minimum diagnostic duration and count level. This strategy should be interpreted as a possible rescue approach rather than as a standardized alternative to full-duration acquisition, particularly when reacquisition is not feasible.

The findings of this review support the development of adaptive amyloid PET protocols rather than one-size-fits-all reductions. In stable examinations with clearly positive or clearly negative findings, moderate acquisition shortening may be acceptable under validated local conditions. In examinations at increased risk of technical degradation, shorter acquisition may improve tolerability and reduce the opportunity for motion, but it should be combined with quality-control documentation, semiquantitative support, and cautious visual interpretation [27,28,30,40]. By contrast, aggressive shortening, extreme count reduction, borderline amyloid burden, low reader confidence, treatment-eligibility assessment, longitudinal follow-up, or clinical-trial imaging should favor standard validated acquisition protocols whenever feasible.

In non-repeatable or technically degraded scans, AI-based restoration and motion correction may serve as supportive or rescue tools, but they should not be treated as evidence of diagnostic equivalence to standard acquisition. AI-restored images require awareness of possible artifacts, over-smoothing, domain shift, threshold instability, and altered quantitative calibration. Similarly, motion correction may improve image quality and affect final interpretation, but interpretation changes should be described as diagnostic impact or classification shifts rather than proven accuracy improvement unless an independent reference standard is available [34,35,36,38,39,40].

Future prospective multicenter studies should directly evaluate these strategies in the clinical scenarios for which they are intended, including motion-prone, poorly cooperative, interrupted, low-count, and non-repeatable acquisitions. Such studies should compare conventional and digital platforms using the same tracer, injected activity, uptake window, reconstruction strategy, and reporting endpoint. They should separately report visual confidence, reader agreement, SUVR and Centiloid stability, borderline behavior, threshold calibration, motion magnitude, motion-correction effects, longitudinal reproducibility, and clinical reporting safety [20,21,22,23,30,33,40,47].

The future framework is therefore not a universal reduced protocol, but a tracer-, scanner-, acquisition-, and task-specific optimization strategy guided by acquisition quality, quantitative calibration, motion control, and diagnostic risk, and local clinical governance.

## 5. Conclusions

Moderate reductions in acquisition time or effective counts appear technically feasible for amyloid PET with [^18^F]florbetaben, [^18^F]florbetapir, and [^18^F]flutemetamol in selected settings, particularly when visual interpretation is supported by semiquantitative assessment.

However, the current evidence does not support a universal reduced protocol across tracers, scanners, reconstruction methods, acquisition windows, or clinical tasks. Shortened or reduced-count protocols require tracer-, scanner-, acquisition-, and task-specific validation, and quantitative thresholds may need recalibration when acquisition conditions change.

The evidence base remains uneven across technical domains. Acquisition-time reduction currently has the strongest amyloid PET-specific support, whereas injected-activity reduction and simulated low-count imaging remain partly dependent on list-mode or modeled data. AI-based restoration is promising for short, low-count, or non-repeatable acquisitions, but remains model-specific and requires external validation. Motion correction directly addresses head-motion-related spatial degradation, but amyloid-specific evidence is currently limited to one [^18^F]flutemetamol PET/CT study; changes in final interpretation should therefore be described as diagnostic impact or classification shifts rather than proven accuracy improvement.

Reduced acquisition protocols, AI-based restoration, and motion correction should be interpreted as supportive or rescue-oriented strategies rather than substitutes for validated standard acquisition. Standard protocols remain preferable in borderline amyloid burden, low reader confidence, treatment-eligibility assessment, longitudinal follow-up, and clinical-trial imaging whenever feasible.

Future amyloid PET optimization should move toward adaptive, task-specific protocols guided by image quality, motion control, quantitative stability, and diagnostic risk.

## Figures and Tables

**Table 1:** Studies evaluating acquisition-time reduction.

Authors	Tracer/Population	Injected Activity and PET System	Strategy and Comparator	Main Clinically Relevant Finding
Tiepolt et al., 2013 [25]	[^18^F]florbetaben; *n* = 50 participants (25 AD, 25 healthy controls; quantitative subset: 10 AD, 10 healthy controls).	~300 MBq; CTI ECAT EXACT HR+ (CTI/Siemens, Knoxville, TN, USA)	Retrospective reconstruction of 5, 10, and 20 min acquisitions	Five min imaging preserved group discrimination, visual classification, reader confidence, and inter-reader agreement under the tested conditions.
Otani et al., 2021 [26]	[^18^F]flutemetamol; *n* = 15 participants (7 Aβ-positive, 8 Aβ-negative).	~185 MBq; Discovery PET/CT 710 (GE Healthcare, Waukesha, WI, USA)	Comparison of 10, 20, and 30 min acquisitions and different scan-start timings	Visual diagnosis was unchanged, but SUVR was sensitive to acquisition duration and scan-start timing, especially in Aβ-positive scans.
Wagatsuma et al., 2022 [27]	[^18^F]florbetapir; *n* = 40 participants (14 Aβ-positive, 26 Aβ-negative).	~370 MBq; Discovery MI PET/CT and Discovery PET/CT 710 (GE Healthcare, Waukesha, WI, USA)	Retrospective reconstruction of 5, 10, and 20 min acquisitions	Quantitative accuracy and inter-acquisition agreement were largely preserved with shortened acquisitions.
Xie et al., 2023 [28]	[^18^F]florbetapir; *n* = 42 participants (29 AD, 13 healthy controls).	3.7 MBq/kg; SIGNA PET/MR 3T (GE Healthcare, Waukesha, WI, USA)	PET/MR reconstruction from 1 to 15 min acquisitions	Diagnostic performance was preserved down to 6 min, whereas visual and quantitative reliability declined with more aggressive shortening.
Trinh et al., 2024 [29]	[^18^F]florbetaben; *n* = 307 participants (199 Aβ-positive, 108 Aβ-negative).	~300 MBq; Discovery STE PET/CT (GE Healthcare, Waukesha, WI, USA)	Retrospective reconstruction of 5, 10, 15, and 20 min acquisitions	Five min imaging showed stable SUVR and Centiloid values, but acquisition-specific threshold calibration may be needed.
Yang et al., 2024 [30]	[^18^F]florbetapir; *n* = 38 participants (29 Aβ-positive, 9 Aβ-negative).	~300 MBq; uMI Panorama PET/CT (United Imaging Healthcare, Shanghai, China)	Ultra-fast imaging from 30 s to 10 min on a high-performance digital PET/CT system	Very short acquisitions were technically feasible on a high-performance scanner, but visual specificity decreased at the shortest durations.

**Table 2:** Studies evaluating injected-activity reduction or simulated low-count imaging.

Authors	Tracer/Population	Injected Activity and PET System	Strategy and Comparator	Main Clinically Relevant Finding
Joshi et al., 2012 [31]	[^18^F]florbetapir; *n* = 20 participants (9 AD, 11 cognitively normal controls); test–retest cohort: *n* = 20 participants (10 AD, 10 cognitively normal controls).	111 versus 370 MBq; PET system not reported	Real low- versus standard-dose comparison	Lower injected activity preserved SUVR and visual classification in the tested cohort, with slightly reduced image quality.
Herholz et al., 2014 [32]	[^18^F]florbetapir; *n* = 4 participants (2 AD, 2 healthy controls), with modeled cohort analysis.	~300 MBq; HRRT (CTI PET Systems, Knoxville, TN, USA)	Simulated dose reduction to 50%, 20%, and 10% of original counts	Region-based amyloid classification was relatively robust, although uncertainty increased at lower count levels.
Young et al., 2024—FMM [33]	[^18^F]flutemetamol; *n* = 175 participants (81 Aβ-positive, 94 Aβ-negative).	~190 MBq; Biograph 64 mCT, Biograph 128 Edge mCT Flow, and Biograph Vision 600 Edge (Siemens Medical Solutions USA, Inc., Knoxville, TN, USA)	Simulated dose reduction to 75%, 50%, 25%, 12.5%, and 5% of original counts	Quantitative and visual performance were generally preserved across simulated reductions, but translation to real low-count conditions requires validation.
Young et al., 2024—FBB [33]	[^18^F]florbetaben; *n* = 75 participants (9 Aβ-positive, 66 Aβ-negative).	~300 MBq; Biograph 4 Emission Duo LSO and TruePoint HiRez (Siemens Medical Solutions USA, Inc., Knoxville, TN, USA)	Simulated dose reduction to 75%, 50%, 25%, 12.5%, and 5% of original counts	Quantitative performance was preserved in most conditions, but agreement decreased in grey-zone cases, supporting caution in borderline scans.

**Table 3:** Studies evaluating AI-based image enhancement or restoration.

Authors	Tracer/Population	Injected Activity and PET System	Strategy and Comparator	Main Clinically Relevant Finding
Chen et al., 2019 [34]	[^18^F]florbetaben; 40 PET/MR datasets from 39 participants.	~330 MBq; GE SIGNA PET/MR	CNN restoration of simulated 1% low-dose PET using PET-only or PET + MR inputs	PET + MR-based deep learning markedly improved image quality and amyloid-status classification compared with raw low-count PET.
Chen et al., 2021 [35]	[^18^F]florbetaben; *n* = 18 participants (4 AD, 6 MCI, 8 healthy controls), plus 32 pretraining cases.	Ultra-low-dose activity 6.6 ± 3.6 MBq; standard dose 300 MBq; SIGNA PET/MR (GE Healthcare, Waukesha, WI, USA)	Deep-learning enhancement of true ultra-low-dose PET/MR, compared with standard-dose PET	Enhanced ultra-low-dose images became clinically interpretable and showed high agreement with standard-dose PET.
Chen et al., 2022 [36]	[^18^F]florbetaben; *n* = 16 participants (8 healthy controls, 8 MCI/AD), plus 32 pretraining cases.	Ultra-low-dose activity 6.5 ± 3.8 MBq; standard dose 300 MBq; SIGNA PET/MR (GE Healthcare, Waukesha, WI, USA)	Deep-learning enhancement using simultaneous versus non-simultaneous MR input	Enhancement improved ultra-low-dose PET quality with similar performance using simultaneous or non-simultaneous MR information.
Lee et al., 2021 [37]	[^18^F]florbetaben; *n* = 84 participants (22 cognitively normal, 20 MCI, 42 AD).	300 MBq; Discovery 600 PET/CT (GE Healthcare, Waukesha, WI, USA)	CNN prediction of full-time PET/CT from 1, 2, 3, 4, and 5 min short acquisitions	AI-based prediction supported scan-time reduction, with more reliable image quality from 2–3 min onward.
Jeong et al., 2021 [38]	[^18^F]florbetaben; *n* = 294 training/internal-validation participants, *n* = 28 temporal-validation participants, and *n* = 58 external-validation participants.	300 MBq; Biograph mCT Flow PET/CT (Siemens Medical Solutions USA, Inc., Knoxville, TN, USA)	GAN restoration of 2 min PET to synthetic 20 min-like PET	GAN-restored images improved image quality and preserved BAPL-based interpretation, including temporal and external validation.
Peng et al., 2021 [39]	[^18^F]florbetapir; *n* = 25 participants (13 Aβ-positive, 12 Aβ-negative).	370 MBq; PET system not reported	Deep-learning denoising of 1, 2, 5, and 10 min acquisitions using U-Net, MCDNet, and MCDNet-2	MCDNet-based denoising improved image quality and classification compared with raw or conventionally filtered short acquisitions.

**Table 4:** Studies evaluating motion correction.

Authors	Tracer/Population	Injected Activity and PET System	Strategy and Comparator	Main Clinically Relevant Finding
Park et al., 2024 [40]	[^18^F]flutemetamol; 108 participants	185 MBq; Biograph Vision 600 PET/CT (Siemens Medical Solutions USA, Inc., Knoxville, TN, USA)	Data-driven head-motion correction of conventional 20 min PET/CT acquisitions	Motion correction improved image quality and inter-reader agreement and changed final interpretation in 11/108 scans; this should be interpreted as diagnostic impact or classification shift, not proven accuracy improvement.

## Data Availability

Data supporting the reported results can be found using the public scientific databases cited in the text.

## Supplementary Materials

The following supporting information can be downloaded at: https://www.mdpi.com/article/10.3390/diagnostics16132033/s1, Figure S1: Study Selection Flow Diagram; Table S1: Reports excluded; Table S2: Summarizes the domain-level evidence-maturity appraisal.

## Abbreviations

The following abbreviations are used in this manuscript:

AD	Alzheimer’s disease
AI	Artificial intelligence
Aβ	Amyloid beta/beta-amyloid
BAPL	Brain amyloid plaque load
CI	Confidence interval
CNN	Convolutional neural network
COV	Coefficient of variation
cSUVR	Composite standardized uptake value ratio
CT	Computed tomography
DL	Deep learning
GAN	Generative adversarial network
ICC	Intraclass correlation coefficient
MCDNet	Monte Carlo Denoising Net
MCI	Mild cognitive impairment
ML	Machine learning
MR	Magnetic resonance
NPV	Negative predictive value
NRMSE	Normalized root mean square error
PET	Positron emission tomography
PET/CT	Positron emission tomography/computed tomography
PET/MR	Positron emission tomography/magnetic resonance
PMT	Photomultiplier tube
PPV	Positive predictive value
PSNR	Peak signal-to-noise ratio
RMSE	Root mean square error
ROC	Receiver operating characteristic
rSUVR	Regional standardized uptake value ratio
SiPM	Silicon photomultiplier
SSIM	Structural similarity index measure
SUVR	Standardized uptake value ratio
