# Supplementary material for: Technical Optimization Strategies for Amyloid PET Under Challenging Acquisition Conditions: A Comprehensive Narrative Review
Source: Diagnostics (Basel). 2026 Jun 29;16(13):2033. doi: 10.3390/diagnostics16132033 (PMC13359891; doi:10.3390/diagnostics16132033)
Supplement: Supplementary file 1 [file diagnostics-16-02033-s001.zip › diagnostics-4370592-supplementary.pdf]

Supplementary Materials

Figure S1 Study Selection Flow Diagram.

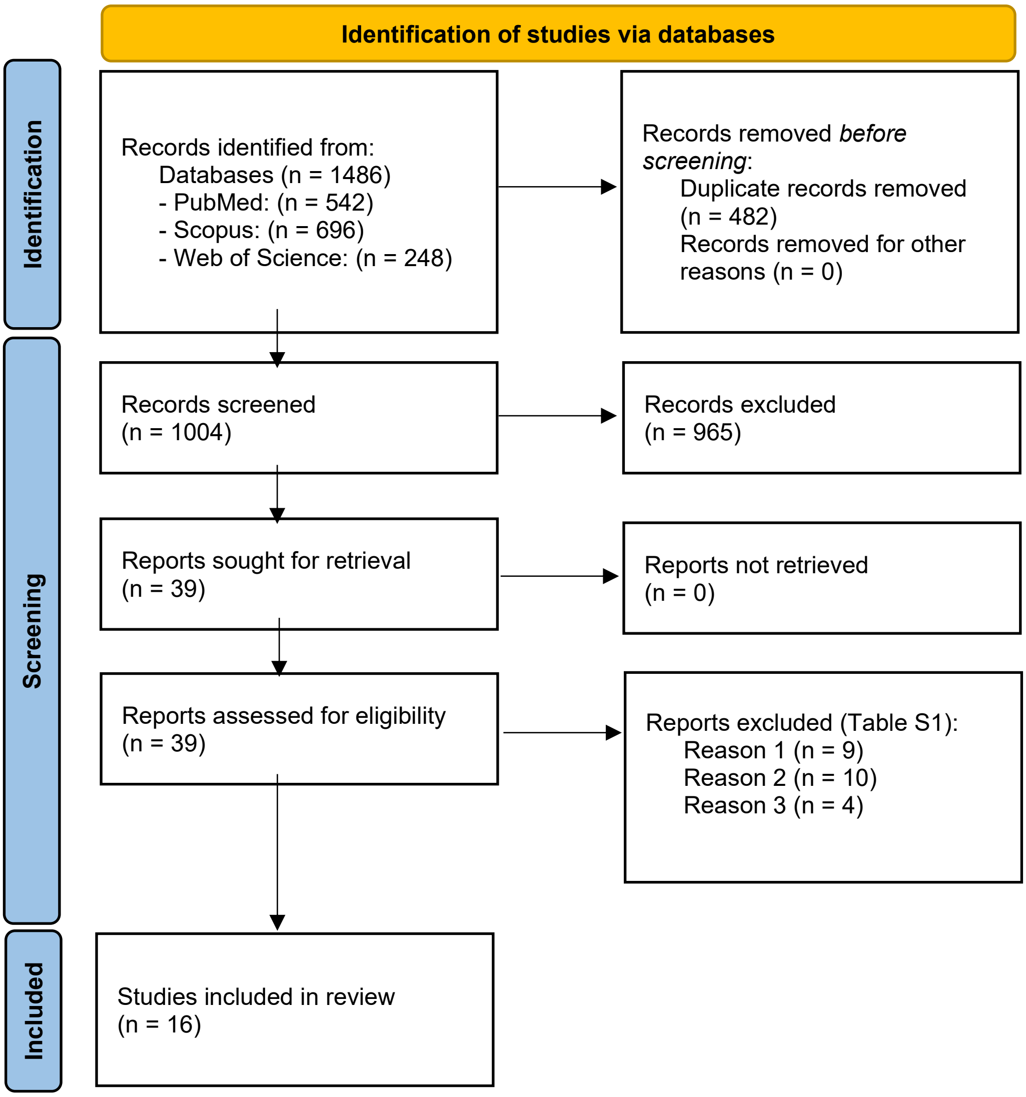

Table S1. Reports excluded.

Reason 1: Early- or middle-phase acquisition windows, alternative time windows, or predictive studies not primarily focused on technical optimization (n = 9)	
1.	Boccalini et al., 2020 – Early-Phase 18F-Florbetaben PET as an Alternative Modality for 18F-FDG PET
2.	Daerr et al., 2016 – Evaluation of early-phase [(18)F]-florbetaben PET acquisition in clinical routine cases
3.	Hsiao et al., 2012 – Correlation of early-phase 18F-florbetapir PET images to FDG images: preliminary studies
4.	Sanaat et al., 2024 – A deep learning model for generating [18F] FDG PET images from early-phase [18F] Florbetapir and [18F] Flutemetamol PET images
5.	Sanaat et al., 2025 – Tracer-Separator: A Deep Learning Model for Brain PET Dual-Tracer (18F-FDG and Amyloid) Separation
6.	Wu et al., 2023 – Feasibility evaluation of middle-phase 18F-florbetaben PET using Centiloid quantification and visual assessment
7.	Johns et al., 2024 – Florbetaben amyloid PET acquisition time: Influence on Centiloids and interpretation
8.	Park et al., 2025 – Machine learning-based prediction of amyloid positivity using early-phase 18F-flutemetamol PET
9.	Choi et al., 2025 – Machine learning model for predicting Aβ positivity and cognitive status using early-phase 18F-florbetaben PET and clinical features
Reason 2: Protocol/software comparison studies without a direct technical optimization intervention or studies involving non-target tracers (n = 10)	
1.	Cheon et al., 2025 – Comparison of Amyloid-PET Analysis Software Using 18F-Florbetaben PET in Patients with Cognitive Impairment
2.	Kang et al., 2025 – Reliability of Automated Amyloid PET Quantification: Real-World Validation of Commercial Tools Against Centiloid Project Method

3.	Zeltzer et al., 2025 – Concordance Between Amyloid-PET Quantification and Real-World Visual Reads
4.	Bourgeat et al., 2026 – AI-enhanced Centiloid quantification of amyloid PET images
5.	Cody et al., 2025 – Comparison of amyloid PET acquired through standardized and unstandardized protocols
6.	Li, B. et al., 2022 – A cross-scanner and cross-tracer deep learning method for the recovery of standard-dose imaging quality from low-dose PET.
7.	Yamada et al., 2025 – Crossover evaluation of time-of-flight-based attenuation correction in brain 18F-FDG and 18F-flutemetamol PET
8.	Ruwanpathirana et al., 2024 – Impact of PET Reconstruction on Amyloid-β Quantitation in Cross-Sectional and Longitudinal Analyses
9.	Ikari et al., 2016 – Phantom criteria for qualification of brain FDG and amyloid PET across different cameras
10.	Odagiri et al., 2024 – Verification of the effect of data-driven brain motion correction on PET imaging
Reason 3: Studies focused primarily on the CT or MR component rather than amyloid PET optimization (n = 4)	
1.	Kim et al., 2022 – Development and clinical validation of CT-based regional modified Centiloid method for amyloid PET
2.	Matsuda et al., 2021 – Amyloid PET quantification using low-dose CT-guided anatomic standardization (2021)
3.	Kawamura et al., 2026 – Comparison of MRI-, CT- and PET-based anatomical standardization for Centiloid scale calculation in 18F-florbetapir PET
4.	Khalighi et al., 2026 – Enhancing the Diagnostic Accuracy of Amyloid PET: The Impact of MR-Guided PET Reconstruction

Table S2. Summarizes the domain-level evidence-maturity appraisal. Overall, evidence maturity was uneven across technical domains: acquisition-time reduction had the closest support for clinical translation, whereas reduced-count imaging, AI-based restoration, and motion correction remained more dependent on simulated data, model-specific validation, or limited amyloid-specific replication. This pattern supports a cautious interpretation of technical feasibility and reinforces the need for tracer-, scanner-, reconstruction-, and task-specific validation before protocol modification.

Technical domain	Strength of evidence	Clinical readiness	Key validation gaps
**Acquisition-time reduction**	Moderate. Supported by several amyloid PET studies across the main [^{18}F]-labeled tracers, with visual and semiquantitative endpoints generally preserved under moderate shortening.	Most clinically mature domain. Moderate shortening may be considered when standard acquisition is limited by tolerance, discomfort, or motion risk, provided local tracer-, scanner-, and reconstruction-specific validation is performed.	No universal minimum scan duration can be defined. Ultra-short protocols remain scanner-dependent, and prospective validation in motion-prone, poorly compliant, interrupted, or borderline cases is limited.
**Injected-activity reduction or simulated low-count imaging**	Low to moderate. Moderate count reduction appears quantitatively robust, but much of the evidence comes from simulated or list-mode reduced-count analyses.	Potentially useful for interpreting low-count or technically compromised studies, including decay, extravasation, delayed acquisition, partial acquisition, or non-repeatable scans. Routine intentional dose reduction is not yet sufficiently validated.	Reduced activity, reduced counts, and shortened acquisition are related but not interchangeable. Borderline cases, visual confidence, local thresholds, and reconstruction-dependent effects require further study.
**AI-based image enhancement or restoration**	Early to moderate. Several amyloid-specific studies show improved image quality, quantitative agreement, and amyloid-status classification after AI restoration, but results remain model-specific.	Promising as a supportive or rescue-oriented post-processing strategy for short, low-count, or non-repeatable acquisitions. It should not currently replace validated acquisition protocols.	External multicenter validation remains incomplete. Generalizability across tracers, scanners, reconstruction pipelines, acquisition windows, and amyloid-burden distributions is uncertain.
**Motion correction**	Low but clinically relevant. Evidence is limited to one amyloid-specific [^{18}F] flutemetamol PET/CT study, but it directly addresses head motion during acquisition.	Potentially useful when motion is present or when reacquisition is not feasible. It is complementary to acquisition shortening and AI restoration.	Evidence is limited to one tracer, scanner platform, reconstruction method, and retrospective dataset. Interpretation changes should be reported as diagnostic impact or classification shifts, not proven accuracy improvement.
